# Risk factors and incidence of invasive bacterial infection in severe bronchiolitis: the RICOIB prospective study

**DOI:** 10.1186/s12887-022-03206-4

**Published:** 2022-03-17

**Authors:** Carmina Guitart, Carme Alejandre, Sara Bobillo-Perez, Monica Girona-Alarcon, Anna Sole-Ribalta, Francisco Jose Cambra, Monica Balaguer, Iolanda Jordan

**Affiliations:** 1 Pediatric Intensive Care Unit. Hospital Sant Joan de Déu, University of Barcelona, 2 Esplugues de Llobregat, 08950 Barcelona, Spain; 2 Immunological and Respiratory Disorders in the Paediatric Critical Patient Research Group. Research Institute of Sant Joan de Déu, University of Barcelona, Barcelona, Spain; 3 Pediatric Infectious Diseases Research Group, Research Institute of Sant Joan de Déu CIBERESP, Barcelona, Spain

**Keywords:** Bronchiolitis, Viral, Bacterial Infections, Outcomes, Risk Factors

## Abstract

**Background:**

Bacterial infection (BI), both community-acquired (CA-BI) and hospital-acquired (HAI), might present as a severe complication in patients with bronchiolitis. This study aimed to describe BI in children with severe bronchiolitis, and to define risk factors for BI.

**Methods:**

This was a prospective, descriptive study that included infants admitted to the pediatric intensive care unit (PICU) due to bronchiolitis between 2011 and 2017. The BROSJOD score was calculated to rate the severity of bronchiolitis.

**Results:**

Inclusion of 675 patients, with a median age of 47 days (IQR 25–99). 175 (25.9%) patients developed BI, considered HAI in 36 (20.6%). Patients with BI had higher BROSJOD score, PRISM III, and required invasive mechanical ventilation and inotropic support more frequently (p < 0.001). BI was independently associated with BROSJOD higher than 12 (OR 2.092, 95%CI 1.168–3.748) CA-BI was associated to BROSJOD > 12 (OR 2.435, 95%CI 1.379–4.297) and bacterial co-infection (OR 2.294 95%CI 1.051–5.008). Concerning HAI, an independent association was shown with mechanical ventilation longer than 7 days (OR 5.139 95%CI 1.802–14.652). Infants with BI had longer PICU and hospital stay (*p* < 0.001), Mortality was higher in patients with HAI.

**Conclusions:**

A quarter of infants with severe bronchiolitis developed BI. A BROSJOD > 12 may alert the presence of CA-BI, especially pneumonia. Patients with BI have higher morbidity and mortality.

**Supplementary Information:**

The online version contains supplementary material available at 10.1186/s12887-022-03206-4.

## Background

Acute bronchiolitis is the most common viral infection of the lower respiratory tract in infants [1–3], defined as the first episode of respiratory distress preceded by a catarrh, in children under 2 years of age.

Of the estimated children under the age of 5 years who require care for RSV infections annually, 78% are over the age of 1 year [4]. Although the majority of children with bronchiolitis do not require hospitalization, approximately 3% of them are admitted, accounting for 18% of total hospital admissions in children under 1 year of age [5]. Between 2–6% of the hospitalized patients, require mechanical respiratory support and admission to the pediatric intensive care unit (PICU), which represents 13% of total PICU admissions. This translates into a high occupancy during the winter season [5]. Prematurity, chronic lung disease, congenital heart disease, and passive exposure to tobacco, among others, are described as risk factors for presenting bronchiolitis requiring hospitalization, but few articles describe risk factors associated with admission to the PICU and its comorbidities [6, 7]. 

Bacterial infection (BI) is a serious, although rare complication in patients with acute bronchiolitis who require PICU admission. Community-acquired BI (CA-BI), which includes bacterial pneumonia, sepsis, urinary tract infection (UTI) meningitis and invasive enteritis, is a relevant cause of morbidity and mortality in PICU [8]. Between 17.5% and 44% [9] of patients with severe bronchiolitis who require admission to the PICU show bacterial growth in respiratory samples from the lower airway (colonization) with a higher risk of developing bacterial pneumonia [6, 10]. Moreover, patients with severe bronchiolitis may develop hospital-acquired infection (HAI) due to mechanical ventilation or vascular and urinary catheters [9]. Health-care acquired pneumonia (HCAP) may occur up to a third of children requiring invasive ventilation, which is associated with longer PICU stay and empirical antibiotic indication [11].

Furthermore, bronchiolitis may trigger a systemic inflammatory response syndrome, with an increase in acute phase reactants, difficult to differentiate from BI, a fact that leads to the indiscriminate administration of antibiotic therapy, especially in children under 3 months of life [12–14]. Currently, there is an increase interest to understand the complex interaction between different characteristic of the host, its microbiota and the virus, and how this interaction may affect the pathogenesis and severity of this disease [15].

The main objectives of this study were to describe BI in patients with severe acute bronchiolitis who required PICU admission, to analyze possible risk factors for developing BI, and to evaluate the main outcomes associated to BI.

## Methods

A descriptive, prospective and observational study was designed. Patients with severe bronchiolitis admitted to the PICU of a referral tertiary hospital (Sant Joan de Déu Hospital, Barcelona) between January 2011 and July 2017, were included. The severity of the bronchiolitis was based on the current definitions [16, 17]. Those patients who had received antibiotic therapy for more than 24 h prior to the PICU admission, and patients with chronic immunosuppression were excluded. Written parental informed consent was mandatory and all parents of an eligible patient agreed to participate in this study.

The following variables were determined: age, sex, medical history and previous comorbidities, Bronchiolitis Score of Sant Joan de Déu (BROSJOD) [18] and Pediatric Risk Score of Mortality III scale (PRISM III) at admission, the treatment administrated (nebulized and endotracheal), viral determination in respiratory sample, the performance or not of bacterial cultures (nasopharyngeal aspirate (NPA) and tracheal aspirate (TA) cultures, or bronchoalveolar lavage (BAL), blood culture, urine culture, cerebrospinal fluid (CSF) culture, and stool culture), the isolated microorganism in these cultures, the final diagnosis of BI, both CA-BI and HAI, the length of PICU and overall hospitalization stay (LOS), the need and duration of the respiratory support including non-invasive ventilation (NIV, meaning CPAP and BiPAP) and conventional mechanical ventilation (CMV), nitric oxide (NO), the length of invasive devices (central line, urinary catheter) utilization, the hemodynamic support and extracorporeal membrane oxygenation (ECMO). Mortality was registered considering any death occurring during the PICU admission. Viral co-infection was considered as the detection of more than one virus in the respiratory sample. The term bacterial co-infection refers to the isolation of more than one bacterium in the TA/BAL.

### Sample collection and processing

The viral determination of the etiological microorganisms was carried out in a NPA or in a TA/BAL (in intubated patients) with the multiplex PCR technique: DNA amplification technique using the polymerase chain reaction with identification of multiple viruses such as respiratory syncytial virus (RSV), rhinovirus, metapneumovirus, influenza and parainfluenza virus, adenovirus, coronavirus, enterovirus, as well as *Bordetella* determination).

Cultures were indicated according to medical criteria when BI was suspected. To differentiate between colonization and infection, the quantification of colony forming units (CFU) per milliliter was used. The TA/BAL culture was considered positive if there was growth of > 10^5^ CFU/mL for TA [19], and > 10^4^ CFU/mL for BAL [20]. Blood culture was considered positive when microorganisms were isolated. Urine culture obtained by catheterization was considered positive when there was growth of > 10^4^ CFU/ml and > 10^5^ CFU/ml when collected by bag [21]. CSF culture was considered positive when microorganisms were isolated. Bacterial growth in the cultures that were finally determined as contaminants according to medical criteria (considering clinical-analytical evolution, the isolated microorganisms and their growth time), were not considered as bacterial infections.

### Definitions

The definitions of CA-BI, included bacterial pneumonia, sepsis, UTI, meningitis, and invasive enteritis. HAI definitions were based on CDC’s criteria diagnosis [11]. HAI included catheter associated blood stream infection (CLABSI), HCAP and catheter associated urinary tract infection (CAUTI). HAI were diagnosed at any point during the PICU admission.

The initiation of antibiotic therapy was indicated following the criteria of the responsible physician according with the clinical and analytical condition of the patient.

### Endpoints

The main endpoint was the presence of bacterial infection. Secondary outcomes were differentiating between CA-BI and HAI, analyzing risk factors for developing each specific bacterial infection, mortality during the PICU admission, and the PICU and hospital LOS.

### Statistical analysis

The categorical data were expressed as absolute and relative rates, while the continuous data were defined as median and interquartile range (IQR) as appropriate. The comparison between qualitative variables was made using the χ2-square test and quantitative variables using the Mann–Whitney's U test. A multivariate logistic regression was used to assess the association between biomarkers and primary endpoint. Variables incorporated into the multivariate model were those with a possible risk association in the univariate analysis. All these results were expressed as odds ratio (OR) and 95% confidence interval (CI), and represented as a natural logarithm in the forest plot using GraphPad Prism version 8 for Windows (GraphPad Software, La Jolla California USA, www.graphpad.com). A *p*-value < 0.05 was considered significant. Statistical analysis was performed using the SPSS®25 program.

*Ethical considerations.*

This study was approved by the institutional Clinical Research Ethics Committee (ART-03–09), and done in compliance with the Declaration of Helsinki.

## Results

**Fig. 1:**
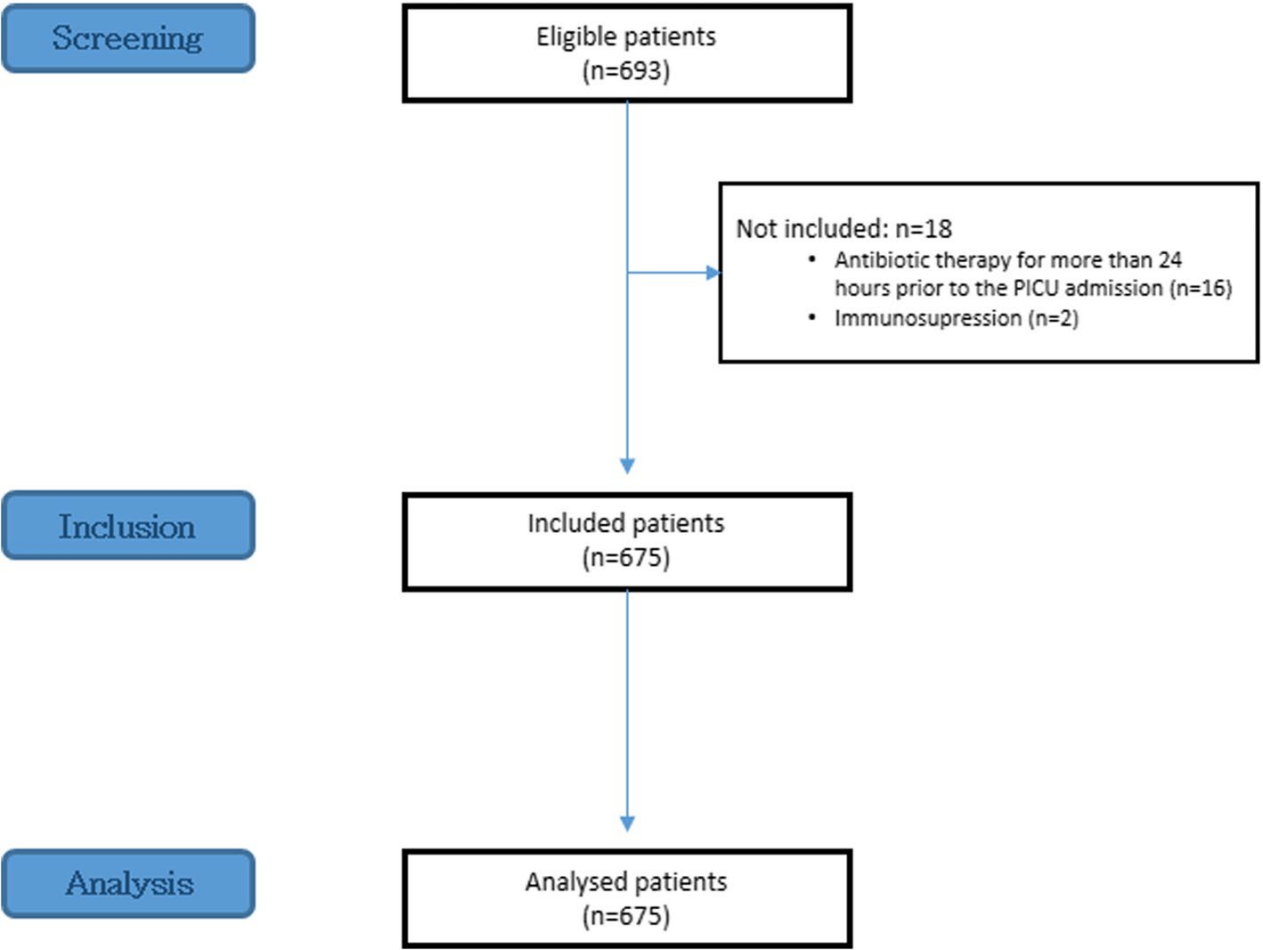
Flow chart

During the period of study, 7,911 patients were admitted to the PICU, 693 (8.8%) with a severe bronchiolitis. The flow diagram is included in Fig. 1. A total of 675 patients were finally included, 59.1% (399 patients) were males. The median age at admission was 47 days (IQR 25–99). The median BROSJOD severity scale at admission was 9 points (IQR 7–11). Other demographic data is expressed in Table 1.

**Table 1:** Demographic and clinical data, including the comparison between No-IBI group and BI group

Variables	Total (*n* = 675)	No-BI (*n* = 500)	BI (*n* = 175)	*p* value
Gender (male), n (%)	399 (59.1)	303 (60.6)	96 (54.9)	0.184
Age (days)	47 (25–99)	48.5 (25–100.8)	44 (24–88)	0.556
< 3 months of age, n (%)	487 (72.1)	355 (71)	132 (75.4)	0.261
Previous comorbidities, n (%)	222 (32.9)	165 (33)	57 (32.6)	0.917
Prematurity	159 (23.6)	119 (23.8)	40 (22.9)	0.800
Respiratory	20 (3)	18 (3.6)	2 (1.1)	0.122
Cardiac	52 (7.7)	37 (7.4)	15 (8.6)	0.617
Neurological	16 (2.4)	10 (2)	6 (3.4)	0.285
Others	36 (5.3)	24 (4.8)	12 (6.9)	0.297
BROSJOD, median (IQR)	9 (7–11)	9 (7–11)	10 (8–12)	0.001
PRISM, median (IQR)	0 (0–3)	0 (0–3)	3 (0–6)	< 0.001
Viral etiology, n (%)				
RSV	445 (65.9)	336 (67.2)	109 (62.3)	0.238
Rhino/Enterovirus	168 (24.9)	121 (24.2)	41 (23.4)	0.837
Metapneumovirus	27 (4)	21 (4.2)	6 (3.4)	0.654
Influenza	32 (4.7)	26 (5.2)	6 (3.4)	0.343
Viral co-infection, n (%)	150 (22.2)	119 (23.8)	31 (17.7)	0.096
LOS, median days (IQR)				
PICU	6 (4–11)	6 (4–9.8)	9 (5–13)	< 0.001
Hospitalization	8 (12–19)	11 (8–19)	14 (9–20)	0.003
Antibiotherapy, n (%)	542 (80.3)	371 (74.2)	171 (97.7)	< 0.001
Respiratory support				
CMV, n (%)	241 (35.7)	136 (27.2)	105 (60.0)	< 0.001
Days, median (IQR)	7.9 (5.5–10.6)	8 (5.6–10.3)	7.9 (5.4–10.9)	1.000
NIV, n (%)	627 (92.9)	463 (92.6)	164 (93.7)	0.622
Days, median (IQR)	3.3 (2–5.1)	3.3 (2.1–4.9)	3.3 (1.9–4.9)	0.521
Inotropic support, n (%)	104 (15.4)	46 (9.2)	58 (33.1)	< 0.001
ECMO, n (%)	6 (0.9)	3 (0.6)	3 (1.7)	0.183
Mortality, n (%)	9 (1.3)	5 (1)	4 (2.3)	0.248

*BI* bacterial infection, *BROSJOD* bronchiolitis score of Sant Joan de Déu, *PRISM* pediatric risk of mortality score, *LOS* length of stay, *PICU* pediatric intensive care unit, *CMV* conventional mechanical ventilation, *NIV* non-invasive ventilation, *ECMO* extracorporeal membrane oxygenation, *RSV* respiratory syncytial virus

### Viral etiology

Regarding viral etiology, 445/675 (65.9%) cases were due to RSV, followed by rhinovirus in 168/675 (24.9%) patients. Viral co-infection (more than one virus) occurred in 150/675 (22%) cases. All data is shown in Table 1.

### Bacterial infection: CA-BI and HAI

One hundred and seventy-five patients/675 (25.9%) presented a BI, considered HAI in 36/175 patients (20.6% of all the patients with BI). CA-BI infections were: 87/675 (12.8%) pneumonia, 65/675 (9.6%) sepsis, and 22/675 (3.3%) UTI. There was no case of meningitis neither invasive enteritis. There were 19/675 (2.8%) HCAP, 3/675 (0.4%) CLABSI, and 19/675 CAUTI (2.8%). The incidence of pneumonia (CA-BI and HCAP) in all the patients was 106/675 (15.7%), 60/241 (24.9%) regarding patients who required CMV. Seven patients had both CA-BI and HAI during PICU stay.

### Microbiological results

There were performed 198/675 TA/BAL (29.3%), of which 138/198 (69.7%) were positive. This corresponded to 20.4% of all patients and 66.8% of the patients on CMV. The most frequently isolated microorganism was *H. influenzae* in 21/138 cases (15%), presenting as a co-infection in 17/21 cases (80%) with: *M. catarrhalis* in 5 cases, *S. aureus* in 6 cases, *S. pneumoniae* in 5 cases, and with *Klebsiella spp*. in 1 case. The other microorganisms most frequently isolated were *S. aureus* in 7/138 (5%%) cases, and *S. pneumoniae* in 6/138 (4.3%) cases. Thirty-eight patients had more than one isolated bacterium.

Five hundred (74.1%) blood cultures were obtained, resulting 66 (13.2%) positives. The most frequently isolated microorganism was *S. epidermidis* (6 cases, 8.8%) followed by *S.hominis* (2 cases, 2.9%). However, those were considered contaminants. There were taken 377 samples for urine culture of which 41/377 (10.8%) were positive. *E. coli* was the most frequent isolated microorganism in 14/41 (34.1%) patients, followed by *Klebsiella spp* in 6/41 (14.6%) patients. Ninety-one 91 lumbar punctures were done, all CSF cultures were negative. Characteristics and isolated microorganisms of each BI type are represented in the Additional file 1 and 2.

### Risk factors

BI diagnosis was significant associated with higher BROSJOD score at admission, PRISM III (*p* < 0.001). Sex, age, previous medical history, or viral etiology among others were not different between patients with BI and patients without BI. When comparing CA-BI and HAI, only differences with the central line and the urinary catheter duration were found. All results are included in Table 2. The univariate analysis of these risk factors for BI, CA-BI and HAI are included in Table 3.

**Table 2:** Risk factors to develop BI in patients with acute bronchiolitis

Risk factor	No BI (*n* = 500)	CA-BI (*n* = 118)	HAI (*n* = 57)	P1	P2	P3
Male gender^a^	303 (60.6)	64 (54.2)	32 (56.1)	0.206	0.813	0.515
Age in days^b^	57 (27–109)	42 (25–92)	46 (24–74)	0.728	0.721	0.475
< 3 months of age^a^	355 (71)	87 (73.7)	45 (78.9)	0.555	0.452	0.206
Previous comorbidities^a^	165 (33)	32 (27.1)	25 (43.9)	0.218	0.027	0.101
Comorbidity’s types^a^						
Prematurity	119 (23.8)	23 (19.5)	17 (29.8)	0.317	0.127	0.316
Respiratory	18 (3.6)	1 (0.8)	1 (1.8)	0.119	0.547	0.709
Cardiologic	37 (7.4)	8 (6.8)	7 (12.3)	0.816	0.223	0.196
Neurological	10 (2.0)	4 (3.4)	2 (3.5)	0.361	1.000	0.352
Others	24 (4.8)	6 (5.1)	6 (10.5)	0.897	0.182	0.070
BROSJOD#	9 (7–11)	10 (8–12)	10 (7.3–11)	< 0.001	0.184	0.296
BROSJOD > 12^a^	36 (7.5)	20 (19.8)	5 (10.4)	< 0.001	0.152	0.480
PRISM scale^b^	3 (0–6)	3 (0–6)	3 (0–6)	< 0.001	0.513	< 0.001
Viral etiology^a^						
RSV	336 (67.2)	80 (67.8)	29 (50.9)	0.901	0.030	0.014
Rhino/Enterovirus	121 (24.2)	27 (22.9)	14 (24.6)	0.763	0.806	0.952
Metapneumovirus	21 (4.2)	5 (4.2)	1 (1.8)	0.986	0.665	0.716
Influenza	26 (5.2)	3 (2.5)	3 (5.3)	0.219	0.393	1.000
Viral co-infection^a^	119 (23.8)	23 (19.5)	8 (14)	0.317	0.376	0.096
Bacterial co-infection^a^	-	13 (11)	4 (7)	-	0.587	-
Received treatment^a^						
Nebulized treatment	411 (82.2)	94 (79.7)	43 (75.4)	0.521	0.525	0.213
Endotracheal mucolytics	40 (8)	25 (21.2)	17 (29.8)	< 0.001	0.210	< 0.001
Central line^a^	110 (22)	67 (56.8)	34 (59.6)	< 0.001	0.719	< 0.001
Central line, days^b^	9 (7–12.5)	8 (6.3–11)	11.8 (7.3–19)	0.453	0.001	0.003
Urinary catheter^a^	134 (26.8)	65 (55.1)	39 (68.4)	< 0.001	0.092	< 0.001
Urinary catheter, days^b^	8.5 (6.3–10.9)	7.8 (6–10.5)	11 (7.1–16.5)	0.976	0.001	0.001

Comparisons made: P1: no BI vs. CA-BI; P2: CA-BI vs. HAI; P3: no BI vs. HAI

*BI* invasive bacterial infection, *CA-BI* community-acquired invasive bacterial infection, *HAI* hospital-acquired infection, *PRISM* Pediatric Risk Score of Mortality III scale, *CMV* conventional mechanical ventilation

^a^Categorical variable, expressed as frequency (%), and compared using Chi-square test

^b^Continuous variable, expressed as median (interquartile range), and compared using the Mann–Whitney test.

**Table 3:** Univariate analysis exploring the different risk factors for each infection

	BI Odds ratio (95%CI)	CA-BI Odds ratio (95%CI)	HAI Odds ratio (95%CI)
Male, gender	1.266 (0.894–1.792)	1.086 (0.749–1.575)	1.870 (0.951–3.677)
< 3 months of age	1.254 (0.845–1.861)	1.232 (0.809–1.877)	1.637(0.705–3.804)
Previous comorbidities	0.981 (0.679–1.416)	0.816 (0.548–1.216)	2.4 (1.222–4.715)
Comorbidity’s types			
Prematurity	0.949 (0.631–1.427)	0.934 (0.604–1.445)	1.673 (0.817–3.428)
Respiratory	0.310 (0.071–1.348)	0.394 (0.090–1.719)	0.932 (0.121–7.167)
Cardiologic	1.173 (0.627–2.195)	0.639 (0.294–1.389)	3.186 (1.323–7.676)
Neurological	1.740 (0.623–4.859)	1.670 (0.571–4.885)	1.189 (0.153–9.257)
BROSJOD > 12	2.470 (1.428–4.271)	2.500 (1.422–4.396)	2.013 (0.739–5.482)
Viral etiology			
RSV	0.806 (0.563–1.153)	0.953 (0.648–1.400)	0.441 (0.225–0.866)
Rhino/Enterovirus	0.958 (0.639–1.437)	0.862 (0.555–1.337)	1.630 (0.796–3.338)
Metapneumovirus	0.810 (0.321–2.040)	1.037 (0.411–2.618)	0.944 (0.927–0.962)
Influenza	0.647 (0.262–1.600)	0.504 (0.174–1.461)	1.912 (0.554–6.603)
Viral co-infection	0.689 (0.444–1.069)	0.792 (0.500–1.253)	0.837 (0.3599–1.951)
Bacterial co-infection	2.454 (1.263–4.769)	2.519 (1.278–4.964)	0.985 (0.228–4.265)
Received treatment and support			
Nebulized treatment	0.781 (0.510–1.196)	0.868 (0.549–1.372)	0.504 (0.241–1.053)
Endotracheal mucolytic	3.632 (2.261–5.834)	3.234 (1.993–5.246)	5.344 (2.612–10.931)
Inotropic support	4.893 (3.160–7.575)	4.354 (2.796–6.778)	3.882 (1.916–7.865)
CMV	4.015 (2.798–5.760)	3.094 (2.122–4.509)	8.344 (3.596–19.360)
CMV > 7 days	2.867 (1.943–4.231)	1.828 (1.207–2.767)	11.361 (5.335–24.197)
Central line	4.839 (3.352–6.985)	3.683 (2.516–5.391)	8.721 (3.902–19.492)
Central line > 7 days	4.283 (2.912–6.300)	3.050 (2.046–4.548)	9.162 (4.392–19.114)
Urinary catheter	4.001 (2.789–5.739)	3.064 (2.102–4.464)	8.524 (3.673–19.778)
Urinary catheter > 5 days	3.840 (2.669–5.524)	2.715 (1.858–3.967)	10.984 (4.726–25.533)
ECMO	2.890 (0.578–14.45)	1.823 (0.331–10.052)	19.273 (3.746–99.167)

*BI* bacterial infection, *CA-BI* community-acquired invasive bacterial infection *HAI* hospital-acquired infection, *BROSJOD* bronchiolitis score of Sant Joan de Déu, *CMV* conventional mechanical ventilation, *ECMO* extracorporeal membrane oxygenation, *RSV* respiratory syncytial virus

**Fig. 2:**
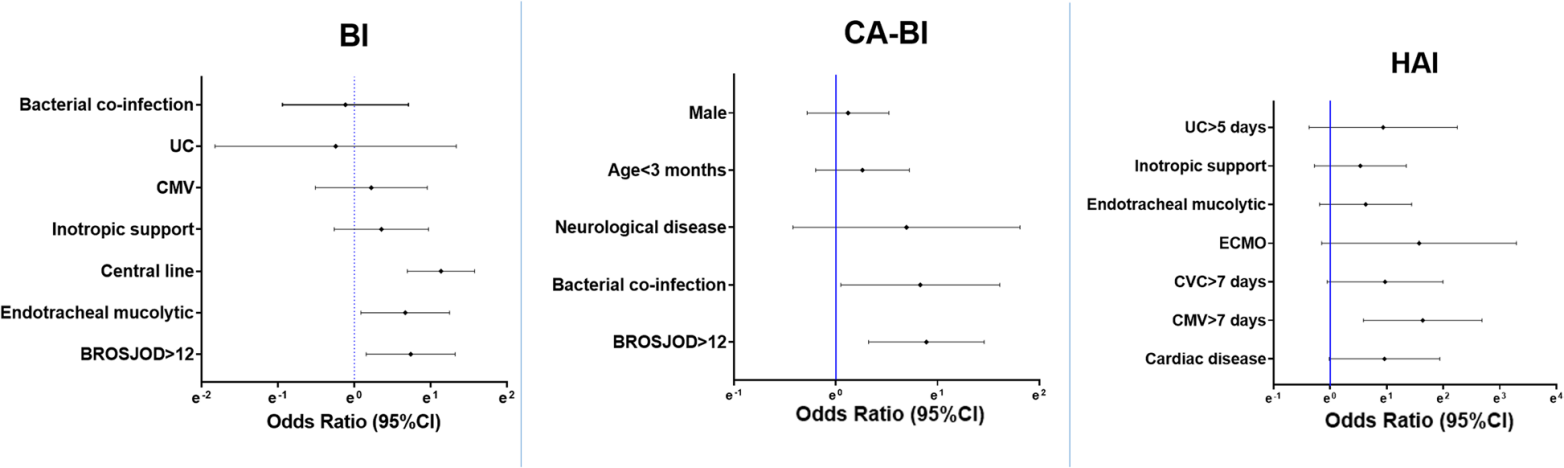
Forest plots representing the multivariate analysis of the risk factors for bacterial infection (BI), community-acquired bacterial infection (CA-BI) and hospital-acquired bacterial infection (HAI). CMV: conventional mechanical ventilation, UC: urinary catheter, ECMO: extracorporeal membrane oxygenation

Independent risk factors for developing a BI were the need for a central line catheter [OR 3.114 (95%CI 2.004–4.839)], a BROSJOD score > 12 points [OR 2.092 (95%CI 1.168–3.748)] and the need for endotracheal mucolytic [OR 1.950 (95%CI 1.091–3.487)], as it is represented in Fig. 2. Regarding CA-BI, the multivariate analysis showed an independent association between CA-BI and a BROSJOD score higher than 12 points at admission [OR 2.435 (95%CI 1.379–4.297)], and the presence bacterial coinfection [OR 2.294 (95%CI 1.051–5.008)], represented in Fig. 2. Additional file 3 includes the forest plot representing the risk factors analysis for the CA-BI subtypes. Finally, HAI was independently associated with CMV for more than 7 days with OR 5.139 (95%CI 1.802–14.652). These results are shown in Fig. 2.

### Morbidity and mortality

Comparing the support received in PICU, no differences were detected between BI and no-BI regarding the use and duration of the NIV, neither when analyzed separately CPAP and BiPAP. However, patients with BI required more frequently inotropic support, CMV and NO. Also the CMV support was longer for HAI. These results are summarized in Table 4.

**Table 4:** Comparison of the respiratory and hemodynamic support received in the PICU, regarding the presence and type of invasive bacterial infection

Support	No BI (*n* = 500)	BI (*n* = 175)	CA-BI (*n* = 118)	HAI (*n* = 57)	P0	P1	P2	P3
Respiratory support								
NIV, n (%)	463 (92.6)	164 (93.7)	110 (93.2)	54 (94.7)	0.622	0.816	0.303	0.726
NIV, days	3.3 (2.1–5.1)	3.3 (1.9–4.9)	3.2 (1.8–4.7)	3.5 (2.2–5.3)	0.521	0.295	0.698	0.554
CMV, n (%)	136 (27.2)	105 (60)	66 (55.9)	39 (68.4)	< 0.001	< 0.001	0.114	< 0.001
CMV, days	8 (5.6–10.3)	7.9 (5.4–10.9)	7.1 (5.2–10.2)	9.8 (6.8–14.7)	1.000	0.090	0.001	0.018
NO, n (%)	2 (0.4)	10 (5.7)	6 (5.1)	4 (7)	< 0.001	0.001	0.730	0.001
Inotropic, n (%)	46 (9.2)	58 (33.1)	39 (33.1)	19 (33.3)	< 0.001	< 0.001	0.970	< 0.001
ECMO, n (%)	3 (0.6)	3 (1.7)	0 (0)	3 (5.3)	0.183	1.000	0.033	0.016

Comparisons made: P0: no BI vs. BI; P1: no BI vs. CA-BI; P2: CA-BI vs. HAI; P3: no BI vs. HAI

*BI* invasive bacterial infection, *CA-BI* community-acquired invasive bacterial infection, *HAI* hospital-acquired infection, *CMV* conventional mechanical ventilation, *NIV* non-invasive ventilation, *NO* nitric oxide,  *ECMO* extracorporeal membrane oxygenation

**Fig. 3:**
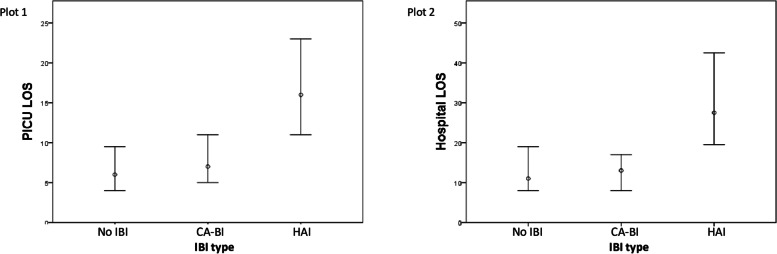
Representation of the median and interquartile range of the length of stay (LOS) for patients without invasive bacterial infection (no BI), patients with community-acquired bacterial infection (CA-BI) and patients with hospital-acquired bacterial infection (HAI). Plot 1 represents the LOS in the pediatric intensive care unit (PICU). Comparison: No BI vs. CA-BI, *p* < 0.001; No BI vs. HAI, p < 0.001; CA-BI vs. HAI, *p* = 0.002. Plot 2 represents the LOS in the hospital. Comparison: No BI vs. CA-BI, *p* = 0.541; No BI vs. HAI, *p* < 0.001; CA-BI vs. HAI, *p* < 0.001

Patients with HAI had longer PICU and hospital LOS than patients with CA-BI and patients without BI, it is represented in Fig. 3. Those patients who had more than one HAI (5, 0.7%) presented higher PICU and hospital LOS: 23 days (IQR 17–38) vs. 6 days (IQR 4–10) with p < 0.001, and 28 days (IQR 23–114) vs. 12 days (IQR 8–19) with *p* = 0.002, respectively. No differences were observed regarding the presence of more than one CA-BI (*n* = 26, 3.9%) for PICU and hospital LOS: More than one CA-BI, 6 days (IQR 5–12) vs. 6 days (IQR 4–10) with *p* = 0.309, and 12 days (IQR 8–19) vs. 12 days (IQR 8–19) *p* = 0.856.

The global mortality was 1.3% (*n* = 9). The highest mortality was observed in patients with HAI (*n* = 2, 5.6%), followed by CA-BI (*n* = 2, 1.4%) and patients without BI (*n* = 5, 1%). No differences were observed considering the presence of more than one HAI (*n* = 1) or CA-BI (*n* = 0).

## Discussion

To our knowledge, this is one of the few articles that analyze risk factors for developing BI in patients with severe bronchiolitis in PICU, and describe morbidity and mortality associated to BI in those patients. Not only for BI, but also for CA-BI and HAI. This study showed that incidence of BI in patients with bronchiolitis is not negligible, being pneumonia the most frequently detected infection. High punctuation in severity scores, should alert physicians that patients may develop BI, which conferred higher morbidity and mortality.

Some studies analyzed the risk of severe bacterial infection development in febrile infants with bronchiolitis, resulting in no significant differences with those without bronchiolitis [22]. In this study, the infection rate was 25.9%. It is essential to differentiate the group of patients who require PICU admission and those patients studied in the emergency room or hospitalization ward. The incidence of BI in the series of patients admitted to PICUs was around 40% [6, 23], whereas in patients in the emergency room or admitted to the hospital ward, varied between 3.5% and 12% [24, 25]. Therefore, the BI diagnosis seemed to confer severity to the patient.

As described in the literature, the most frequently isolated microorganism was *Hemophilus influenza* in up to 52% of cases, alone or in co-infection with other pathogenic microorganisms [14, 26]. Wiegers et al. described bacterial co-infection as a probable cause of longer PICU stay in patients with bronchiolitis [26]. Suárez-Arrabal et al. concluded that those patients with RSV bronchiolitis colonized by gram-negative bacilli were associated with increased severity [27]. By the same token, Diaz-Díaz et al. summarized that viral and host factors were associated with the pathogenesis of RSV disease; and that, recent studies suggested that the complex interaction between the respiratory microbiome, the host's immune response and the virus may have an impact on the pathogenesis and severity of RSV infection. They concluded that the combination of viral-bacterial interaction mechanisms, the association between the microbiota and the severity of RSV infection results in different comorbidity” [15]. Bacterial co-infection was described in more than 1/3 of the patients with bronchiolitis who required invasive mechanical ventilation. Similar than in our results. So, we consider interesting to continue in this research line [26].

It was observed a lower incidence of pneumonia with positive TA/BAL, which occurred in 15.7% of cases, while other studies describe values ​​between 20–45% [14, 23, 26]. This could be due to the fact that these series mostly assess patients with CMV, while our series included all patients, both with invasive and non-invasive ventilation. If we compare these series with our ventilated patients, the values ​​were closer, being 24.9%.

The risk of bacteremia is considered low in children with acute bronchiolitis, with a prevalence between 0.6–2.5% [24], although the occult bacteremia rate is not well established [28]. More recently, in the series by Cebey-López et al*.* bacteremia was detected in 10.6% of their patients [28],. The main isolated microorganisms, according to the literature, were gram-positive cocci such as *S. pneumoniae *[9]. In our series, gram-positive cocci microorganisms were also the principally isolated, although the main ones were *S. epidermidis* and *S. hominis*, in relation to catheter-associated infections. In a smaller percentage, gram negative bacilli such as *Klebsiella* and *Pseudomonas*, were isolated in cases of infection from another origin (UTI).

In multiple studies, UTI was evaluated in febrile patients with mild-moderate acute bronchiolitis not admitted to the PICU. In those patients the infection rate varied between 0% and 5.4% in RSV bronchiolitis [25] compared to 10.1% in non-RSV bronchiolitis [24]. This data was similar to another study that compared the co-infection in bronchiolitis by RSV and non-RSV where urinary infection occurred in 1.3% of positive RSV compared to 10.2% of non-RSV [29]. Our series presented this infection in 3.3% of cases, similar to what is published. The etiology of urinary infections, according to the literature, was mainly due to gram negative bacilli, being the most frequent microorganism *E. coli* [29], as in our series.

As shown in the results and reported by other authors, patients in PICU who developed HAI have considerable higher morbidity, mortality and prolonged hospital stay, and increase health care costs. The patients in ICU are at particularly high risk of infection because of extensive invasive devices and procedures. In this study, the duration of mechanical ventilation and urinary catheter utilization were related to higher HAI development. It could be highlighted the importance of performing the procedures in the best aseptic conditions, and the prompt removal of the devices, to reduce the number of device-associated infections.

Antibiotic treatment in children with bronchiolitis continues to be overused due to the concern of undetected bacterial infection. In our study, the rate of antibiotic prescription was high (80.3%) considering that most of the patients did not have a confirmed bacterial infection. This value was similar to other results in patients admitted to the PICU [13, 30].

As described in the literature, gender, previous co-morbidity, and ethnicity were not related to the severity of children with acute bronchiolitis in PICU [23]. On the other hand, as observed in our results, some authors [5, 28] describe that, patients with acute bronchiolitis and BI tended to be more tachycardic and tachypneic, which were signs of severity and conferred higher score results.. As exposed in the results, a BROSJOD score higher than 12 points at admission, was significant associated with BI, bacterial pneumonia and sepsis.

Regarding morbidity and mortality, infants with BI had longer PICU and overall hospital stay, required more NO, CMV and inotropic support. Some authors [23, 31] described that, patients with bronchiolitis and pneumonia required longer respiratory support than those without BI. However, nasal cannula oxygen therapy, NIV or CMV duration was not found to be related with BI in our series.

The interaction between the host microbiome and viral infection have been presented in recent years as a possible risk factor for severity in patients with acute bronchiolitis, although more studies are still required^24^. It might be interesting to focus on the analysis of the microbiome for future research lines.

The main limitation of this study was that it was performed in a PICU of a single hospital, which may difficult the extrapolation to the general population. However, the large sample size may permit the comparison with a homogeneous population. Moreover, bronchiolitis is a disease that occurs in similar patients and in a certain range of age, so that, it might be possible to compare our population with other series, especially in PICU [1–3]. Secondly, not all the intubated patients had a TA or BAL performed. Finally, the low rate of positive blood cultures in pediatric patients, should be considered, especially if the blood sample is scarce in cases of very young children. Because of this, we decided to consider sepsis by clinical and analytical definition [32, 33], even with negative blood culture. This could modify some of the rates obtained, hence, more studies may be need to confirm the results.

## Conclusions

To sum up, the incidence of BI in patients with bronchiolitis is not negligible. Pneumonia is the most frequently detected infection. Physicians should be concerned about patients with higher punctuation in severity scores, particularly BROSJOD score, who may develop BI, bacterial pneumonia, and sepsis. The length of invasive devices was related to HAI development. Hence, the importance of performing procedures in the best aseptic conditions, and the prompt removal of the devices to reduce HAI. Patients with bronchiolitis that develop BI had higher morbidity and mortality.

## Supplementary Information

**Additional file 1.** Invasive bacterial infection type, including both community-acquired and hospital-acquired infections.

**Additional file 2.** Supplementary material 2. Germen isolation of each invasive bacterial infection type, including community-acquired and hospital-acquired infection.

**Additional file 3.** Forest plots representing the multivariate analysis of the risk factors for the different types of community-acquired bacterial infection: bacterial pneumonia, sepsis, and urinary tract infection.

## Data Availability

The datasets generated and/or analysed during the current study are not publicly available due to lack of informed consent for this use, but are available from the corresponding author upon reasonable request.

## Abbreviations

BAL: Bronchoalveolar lavage
BI: Bacterial infection
BROSJOD: Bronchiolitis Score of Sant Joan de Déu
CA-BI: Community-acquired bacterial infection
CLABSI: Catheter associated blood stream infection
CAUTI: Catheter associated urinary tract infection
CMV: Conventional mechanical ventilation
CSF: Cerebrospinal fluid
ECMO: Extracorporeal membrane oxygenation
HAI: Hospital-acquired bacterial infection
HCAP: Health-care acquired pneumonia
LOS: Length of stay
NPA: Nasopharyngeal aspirate
NIV: Non-invasive ventilation
NO: Nitric oxide
PICU: Pediatric intensive care unit
PRISM III: Pediatric Risk Score of Mortality III scale
RSV: Respiratory syncytial virus
TA: Tracheal aspirate cultures
UTI: Urinary tract infection
