# Supplementary material for: Risk factors and incidence of invasive bacterial infection in severe bronchiolitis: the RICOIB prospective study
Source: BMC Pediatr. 2022 Mar 17;22:140. doi: 10.1186/s12887-022-03206-4 (PMC8926890; doi:10.1186/s12887-022-03206-4)
Supplement: Supplementary file 1 — Additional file 1. Invasive bacterial infection type, including both community-acquired and hospital-acquired infections. [file 12887_2022_3206_MOESM1_ESM.docx]

**Additional file 1.** Invasive bacterial infection type, including both community-acquired and hospital-acquired infections.

|  | Pneumonia (n=106) | Sepsis  (n=68) | UTI  (n=41) | P1 | P2 | P 3 |
| --- | --- | --- | --- | --- | --- | --- |
| Male, n (%) | 52 (49.1) | 40 (58.8) | 26 (63.4) | 0.195 | 0.112 | 0.908 |
| Age (days), median (IQR) | 43 (26-102.8) | 41 (20.5-74.5) | 50 (25-71.5) | 0.069 | 0.524 | 0.253 |
| < 3 months of age, n (%) | 75 (70.8) | 53 (77.9) | 33 (80.5) | 0.245 | 0.091 | 0.558 |
| BROSJOD Score, median (IQR) | 10 (9-12) | 10 (7-12) | 9 (7-11) | 0.134 | 0.035 | 0.663 |
| BROSJOD>12, n (%) | 18 (19.6) | 11 (20.4) | 3 (8.3) | 0.732 | 0.097 | 0.086 |
| LOS (days), median (IQR)  PICU stay  Hospitalization stay | 9 (5-14)  13 (9-23) | 9 (6-14)  15 (10-20) | 8 (5-14.5)  17 (10-26) | 0.353  0.444 | 0.462  0.628 | 0.791  0.289 |
| Respiratory support  CMV, n (%)  Days, median (IQR)  NIV, n (%)  Days, median (IQR) | 60 (56.6)  9.2 (6-12)  100 (94.3)  3.2 (1.7-5) | 45 (66.2)  7.7 (5.3-11.5)  64 (94.1)  3.5 (2-5.5) | 24 (58.5)  8.6 (5.3-14)  38 (92.7)  4.3 (2.4-5.5) | 0.213  0.855  0.847  0.334 | 0.941  0.095  0. 513  0.247 | 0.536  0.973  0.972  0.289 |
| Inotropic support, n, (%) | 30 (28.3) | 35 (51.5) | 12 (29.3) | <0.001 | 0.586 | 0.012 |
| CVC, n (%)  Days, median (IQR) | 60 (56.6)  9.9 (7-14.5) | 48 (70.6)  9.3 (7-12) | 20 (48.8)  10.1 (6.3-18.5) | 0.023  0.821 | 0.241  0.147 | 0.028  0.940 |
| Urinary catheter, n (%)  Days, median (IQR) | 59 (55.7)  9.7 (7-13.8) | 45 (66.2)  8.3 (6.1-11.7) | 24 (58.5)  8.3 (6.2-13.8) | 0.160  0.310 | 0.982  0.048 | 0.536  0.989 |
| ECMO, n (%) | 2 (1.9) | 2 (2.9) | 2 (4.9) | 0.224 | 0.569 | 1.000 |
| Mortality, n (%) | 2 (1.9) | 3 (4.4) | 2 (4.9) | 0.105 | 0.569 | 1.000 |

UTI: urinary tract infection. BROSJOD: bronchiolitis score. PICU: pediatric intensive care unit. LOS: length of stay. CMV: conventional mechanical ventilation. NIV: non-invasive ventilation. CVC: central venous catheter. ECMO: extracorporeal membrane oxygenation.

Comparison made using the Mann-Whitney test. P1 compares pneumonia vs. sepsis; P2 compares pneumonia vs. urinary tract infection; P3 compares sepsis vs. urinary tract infection.
