# Supplementary figures and images for: Risk factors and incidence of invasive bacterial infection in severe bronchiolitis: the RICOIB prospective study
Source: BMC Pediatr. 2022 Mar 17;22:140. doi: 10.1186/s12887-022-03206-4 (PMC8926890; doi:10.1186/s12887-022-03206-4)

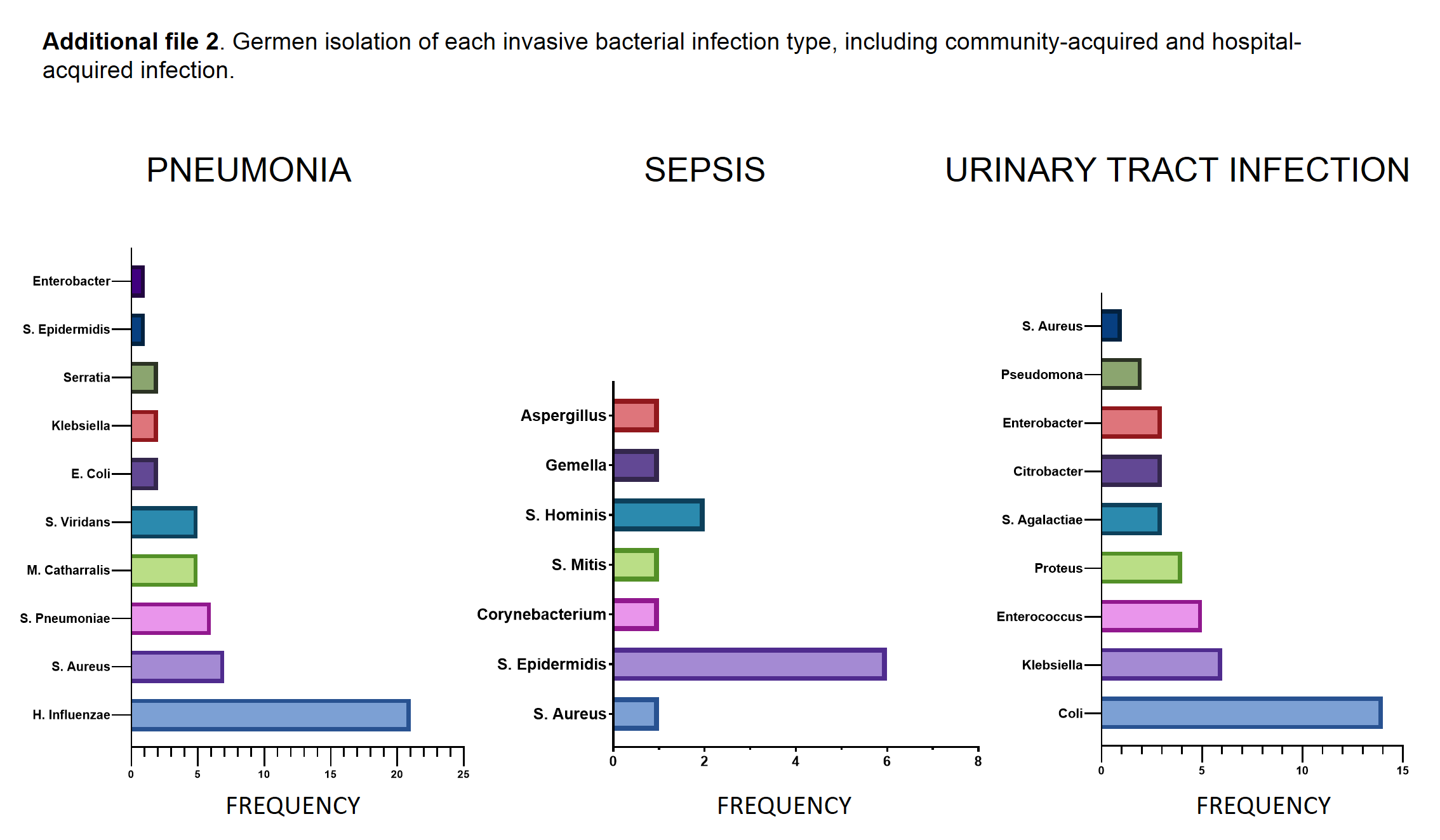

Supplement: Supplementary file 2 — Additional file 2. Supplementary material 2. Germen isolation of each invasive bacterial infection type, including community-acquired and hospital-acquired infection. [file 12887_2022_3206_MOESM2_ESM.tif]

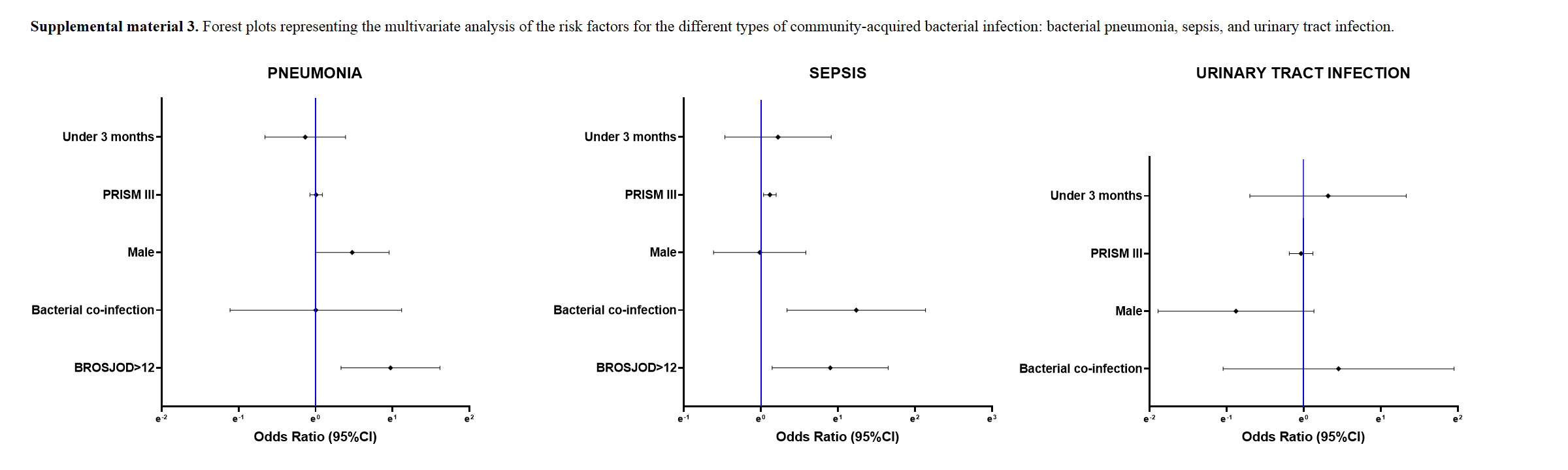

Supplement: Supplementary file 3 — Additional file 3. Forest plots representing the multivariate analysis of the risk factors for the different types of community-acquired bacterial infection: bacterial pneumonia, sepsis, and urinary tract infection. [file 12887_2022_3206_MOESM3_ESM.tif]
